# Fast acquisition protocol for X-ray scattering tensor tomography

**DOI:** 10.1038/s41598-021-02467-w

**Published:** 2021-11-29

**Authors:** Jisoo Kim, Matias Kagias, Federica Marone, Zhitian Shi, Marco Stampanoni

**Affiliations:** 1 Institute for Biomedical Engineering, University and ETH Zürich, 8092 Zurich, Switzerland; 2 Swiss Light Source, Paul Scherrer Institut, 5232 Villigen, Switzerland; 3 Present Address: Division of Engineering and Applied Science, California Institute of Technology, Pasadena, CA 91125 USA

**Keywords:** X-rays, Imaging techniques, Imaging and sensing, Phase-contrast microscopy

## Abstract

Microstructural information over an entire sample is important to understand the macroscopic behaviour of materials. X-ray scattering tensor tomography facilitates the investigation of the microstructural organisation in statistically large sample volumes. However, established acquisition protocols based on scanning small-angle X-ray scattering and X-ray grating interferometry inherently require long scan times even with highly brilliant X-ray sources. Recent developments in X-ray diffractive optics towards circular pattern arrays enable fast single-shot acquisition of the sample scattering properties with 2D omnidirectional sensitivity. X-ray scattering tensor tomography with the use of this circular grating array has been demonstrated. We propose here simple yet inherently rapid acquisition protocols for X-ray scattering tensor tomography leveraging on these new optical elements. Results from both simulation and experimental data, supported by a null space analysis, suggest that the proposed acquisition protocols are not only rapid but also corroborate that sufficient information for the accurate volumetric reconstruction of the scattering properties is provided. The proposed acquisition protocols will build the basis for rapid inspection and/or time-resolved tensor tomography of the microstructural organisation over an extended field of view.

## Introduction

The micro- and nano-structure of various natural and artificial materials is at the base of their macroscopic properties. For instance, the local orientation of collagen fibres is strongly related to the mechanical properties of bone^1,2^ and the local orientation of neuronal axons determines the structural and functional network of the brain^3^. Likewise for synthetic fibre based materials, their mechanical, thermal and electrical properties^4–6^ are dictated by the local fibre orientation as well as by how well the fibres are aligned. The possibility to investigate the microscopic architecture in statistically large enough sample volumes would therefore be valuable during the development of new materials, better understanding existing materials, as well as for product quality control in industrial settings.

X-rays are useful to obtain information on the inner structure of materials in a non-destructive manner. However, in conventional X-ray imaging, just like in many other modalities, a trade-off between spatial resolution and examined field of view (FOV) is unavoidable. With an effective detector pixel size of a few $\upmu \mathrm{m}$ and a few thousands detector pixels, the FOV is limited to a few mm.

The small-angle X-ray scattering (SAXS) signal appears as the Fourier transform of the autocorrelation function of the electron density of a spatially unresolved microstructure. Thus, SAXS contrast can mitigate the trade-off between the length-scale of interest and the FOV, facilitating full correlative studies of the micro- and nano-structure over macroscopic sample volumes^7,8^. The projection image data with local 2D SAXS information can be recorded by scanning a narrow beam corresponding to the size of a single pixel of a projection image data^9^. The 2D SAXS information can be partially recorded by utilising X-ray grating interferometry (XGI), using periodic phase modulating structures creating interference fringe at specific distances downstream^10,11^.

From a collection of projection image data with 2D SAXS information taken at different sample angular poses, we can perform X-ray scattering tensor tomography, to reconstruct the scattering tensor, 3D directional information of microstructure, for each voxel of the reconstructed volume. A set of coefficients of the scattering tensor which models the 3D scattering distribution of the underlying microstructure is reconstructed in each voxel^12–15^.

The acquisition setup needs though multiple tilted rotation axes along the beam direction (two angular degrees of freedom), to provide the necessary information for reconstruction with a higher accuracy^16,17^. This is because anisotropic structures cause anisotropic scattering and the probed signal depends on the relative orientation between the underlying structure and the incident beam^18^. In addition to the two angular degrees of freedom, scanning SAXS based X-ray scattering tensor tomography requires pixel-by-pixel scanning of a narrow beam per projection pose. This requirement intrinsically leads to a long experiment time. X-ray grating interferometry (XGI) based tensor tomography requires additional angular degrees of freedom (in total three) and thus rather complex acquisition geometry^16^ because the linear grating do not provide directional scattering sensitivity, leading to a high acquisition overhead. In addition, XGI based tensor tomography has been exclusively performed on conventional lab-based sources, which requires orders of magnitude longer sample exposure time per projection pose. Therefore, the experiment times of established methods are in the order of several hours hindering any large (cm) scale exploitation of scattering tensor tomography^16,17^.

Circular gratings arranged in an array can provide local small-angle scattering information in multiple directions over the entire FOV in a single shot^19,20^. We have previously demonstrated X-ray scattering tensor tomography with the use of this circular grating array^21^, with which the reduction in experiment time is considerable compared to pre-existing techniques^12,13^ owing to the single-shot omnidirectional scattering sensitivity of the used diffractive optical component. By accelerating the data acquisition, more time-efficient inspection of the microstructure organisation over an extended FOV will be enabled, making this technology attractive for industrial applications as well as for biological and material science studies. To take full advantage of this circular grating array and push the acquisition speed as well as minimise acquisition overhead, different acquisition protocols have to be assessed and investigated.

In this paper, we propose rapid acquisition protocols relying on a setup with two rotational axes and the recently developed circular grating array. It is more challenging to assess how an acquisition geometry will affect the reconstruction accuracy compared to conventional scalar tomography. This is because scattering signals are collected along multiple directions per pixel and the probed directional scattering signal depends on the relative orientation between the underlying structure and the incident beam. Therefore, in the first part, in order to assess and compare the performance of different tensor tomography acquisition protocols, we performed a null space analysis to investigate the intrinsic characteristics of the different acquisition geometries. In the second part, simulation studies are used to validate the acquisition protocols independently from any possible experimental deviations such as sample misalignment and motor inaccuracies. In the last part, experimental studies were finally carried out to test the robustness and confirm the performance of the proposed acquisition protocols also under real investigation conditions.

The proposed rapid acquisition protocols with minimal acquisition overhead coupled with bright X-ray sources will unlock time-resolved X-ray scattering tensor tomography capabilities. The acquisition protocols are potentially compatible and attractive for any other method providing omnidirectional scattering sensitivity in a single shot^22,23^, because the same logic for the null space and the simulation analysis can be applied. The null space analysis framework shown in this manuscript is potentially a good tool to assess acquisition geometry for any tensor tomography method.

## Methods

### X-ray scattering tensor tomography with circular gratings

**Figure 1:**
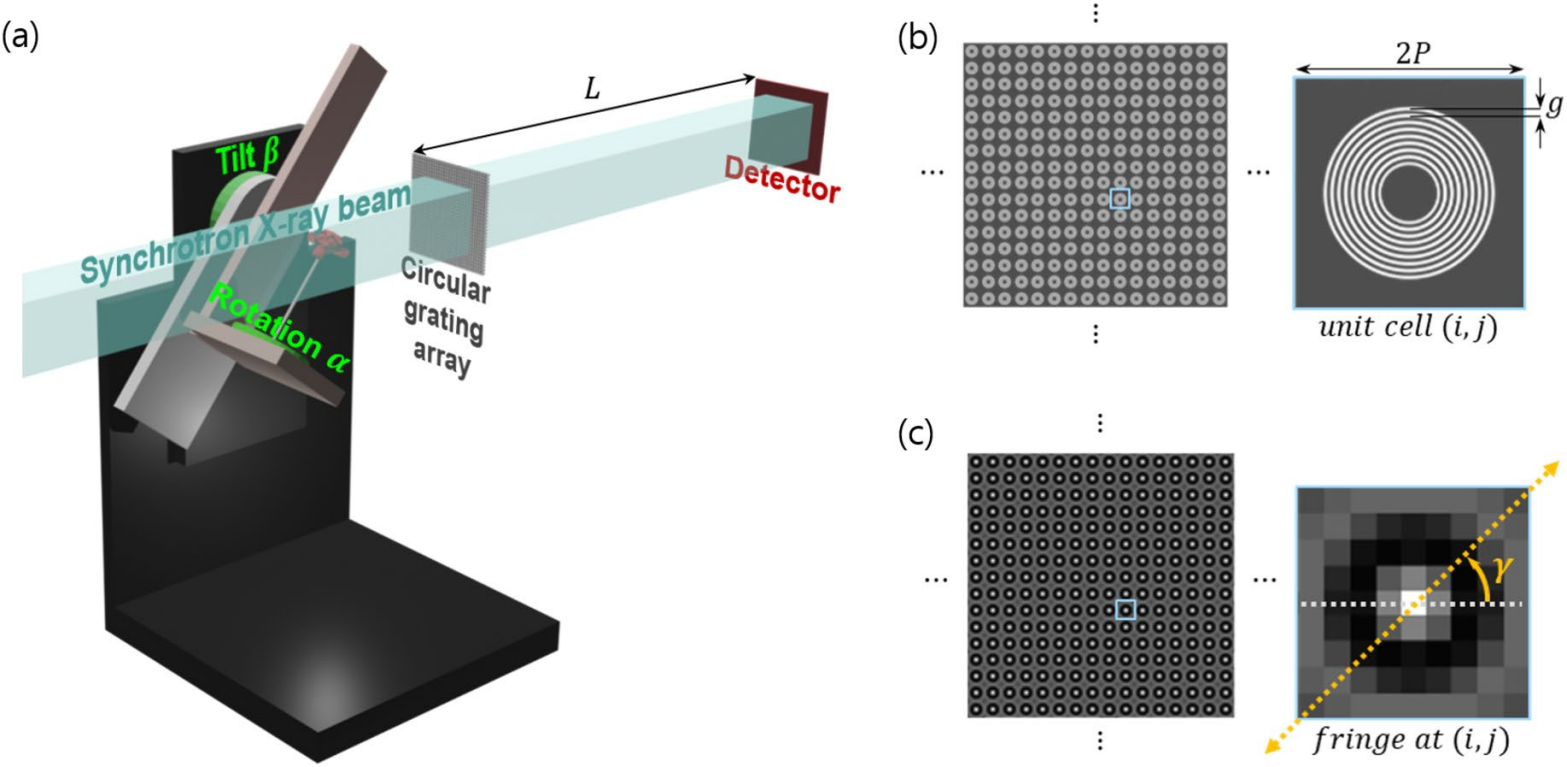
Experimental setup for X-ray scattering tensor tomography with circular gratings. An overview of the setup is shown in (**a**). The rotation motor revolves $\alpha$ degrees and the rotation axis can be tilted $\beta$ degrees. The circular grating array (**b**) is composed of unit cells where *P* is half the unit cell size and *g* is the period of the fine grating lines. It is placed near the sample at a distance $L=Pg/2\lambda$ from the detector. A circular fringe (**c**) is formed at the detector where the 2D directional scattering signal is extracted by measuring the visibility reduction along the radial profile at an angle $-{90}^{\circ }\le \gamma <{90}^{\circ }$.

A schematic overview of the experimental setup for X-ray scattering tensor tomgoraphy with circular gratings is shown in Fig. 1. In order to capture 2D-omnidirectional X-ray scattering signals with a single X-ray projection, a periodic array of multi-circular gratings based on a diffractive annular structure is used in this study^20^. This diffractive structure is characterised by a global period *P* which is half the repetition period of the unit cell and by *g* which is the period of the fine circular gratings within each unit cell as shown in Fig. 1b. The etching depth of the gratings is dependent on the wavelength $\lambda$ of the used X-rays and design phase shift. More details regarding the grating design and fabrication can be found in the reference^20,24^. The circular grating array is placed at a distance $L=Pg/2\lambda$ upstream from the detector as shown in Fig. 1a. The circular diffraction fringe formed at the detector position is readout by multiple detector pixels and an exemplary unit cell window of $9\times 9$ pixels is shown in Fig. 1c. The directional scattering signal is extracted from the fringes recorded on each $9\times 9$ detector pixel grid along different signal extraction angles $\gamma$ (2D). For all studies with the use of circular grating array in this paper, the scattering signal extraction angle $\gamma$ was set to be $\{-{90}^{\circ }, -{67.5}^{\circ }, \ldots , {67.5}^{\circ }\}$. The directional scattering signals are collected from projections acquired with the sample positioned at different angles $\alpha$ and $\beta$. The angle $\alpha$ and $\beta$ represent the rotation and tilt of the stage as shown in Fig. 1a. Then the projection vector $\varvec{p}$ is formed and the elements in $\varvec{p}$ are the measured scattering signals for each $\gamma$, $\alpha$, $\beta$, and detector pixel^21^. The vectorised reconstruction volume $\varvec{\mu }$ is composed of *K* different scattering sampling direction channels $\varvec{\mu }=[\varvec{\mu }_1;\varvec{\mu }_2; \ldots ;\varvec{\mu }_K]$ (3D). Thus, for each reconstruction voxel, the scattering distribution is sampled along *K* different scattering sampling directions. We model the 3D scattering distribution as an ellipsoid, which is expressed as a symmetric rank-2 tensor matrix with 6 independent components. Therefore, at least six scattering sampling directions in 3D ( $K\ge 6$) are required in order to define a unique scattering ellipsoid after reconstruction. Increasing the scattering sampling direction vectors would in general slightly improve the accuracy of the reconstruction; however, this will elongate the reconstruction computation. Therefore, we used seven scattering sampling direction vectors $\varvec{S}_k={[1,0,0], [0,1,0], [0,0,1], [1,1,1]/\sqrt{3}, [-1,1,1]/\sqrt{3}, [1,-1,1]/\sqrt{3}, [1,1,-1]/\sqrt{3}}$ for $k=1,2, \ldots ,7$ for simplicity (three directions parallel to the x, y, and z axes and four diagonal directions). To recover $\varvec{\mu }$, the system of linear equations $A\varvec{\mu }=\sum _{k=1}^{K}V_kW\varvec{\mu }_k=\varvec{p}$ can be solved with any existing solver. *A* is the system matrix computed as a sum of multiplication of the scaling matrices $V_k$ for different scattering sampling directions $\varvec{S}_k$ and the geometric system matrix *W* for discrete beam path integrals^21^. The size of *A* is $M \times N \times K$ where *M* is the number of elements in the projection data, *N* is the number of voxels in the reconstruction volume. Principal component analysis (PCA) is applied to the reconstructed scattering distribution $\varvec{\mu }$ which is modelled with an ellipsoid approximation: three principal axes are computed for each voxel. The principal axis with the shortest length represents the main structure orientation, the mean length of the three principal axes represents the average scattering ($(\lambda _1+\lambda _2+\lambda _3)/3$), and the fractional anisotropy^25^ represents the scattering anisotropy (${\sqrt{(\lambda _1-\lambda _2)^2+(\lambda _2-\lambda _3)^2+(\lambda _3-\lambda _1)^2}}/{2\sqrt{\lambda _1^2+\lambda _2^2+\lambda _3^2}}$), where $\lambda _1,\lambda _2,\lambda _3$ are the eigenvalues of the symmetric 2-rank scattering tensor describing the scattering distribution as an ellipsoid. Detailed information on the signal extraction and tensor reconstruction methods used in this study can be found in the following reference works^12,20,21,26^.

### Acquisition protocols

In general, acquisition protocols which widely and densely cover a virtual unit sphere around samples provide more accurate tensor reconstruction results. It is possible to design optimised but rather complicated trajectories to cover a certain number of scattering orientations, if conventional linear gratings are used^16^. Those complicated trajectories result in a larger acquisition overhead (an effective exposure time divided by the total experiment time) and are not ideal for rapid tensor tomography acquisition. Here, we implement instead two simple acquisition types—stairwise and spiral acquisition protocols, based on a simple two-axis system and compatible with a higher acquisition speed with minimised acquisition overhead. These acquisition protocols rely on the use of circular grating arrays or any other method with omnidirectional scattering sensitivity such as the speckle pattern based^22^ as well as the recently developed omnidirectional simultaneous reciprocal and real space imaging method^27^.

**Figure 2:**
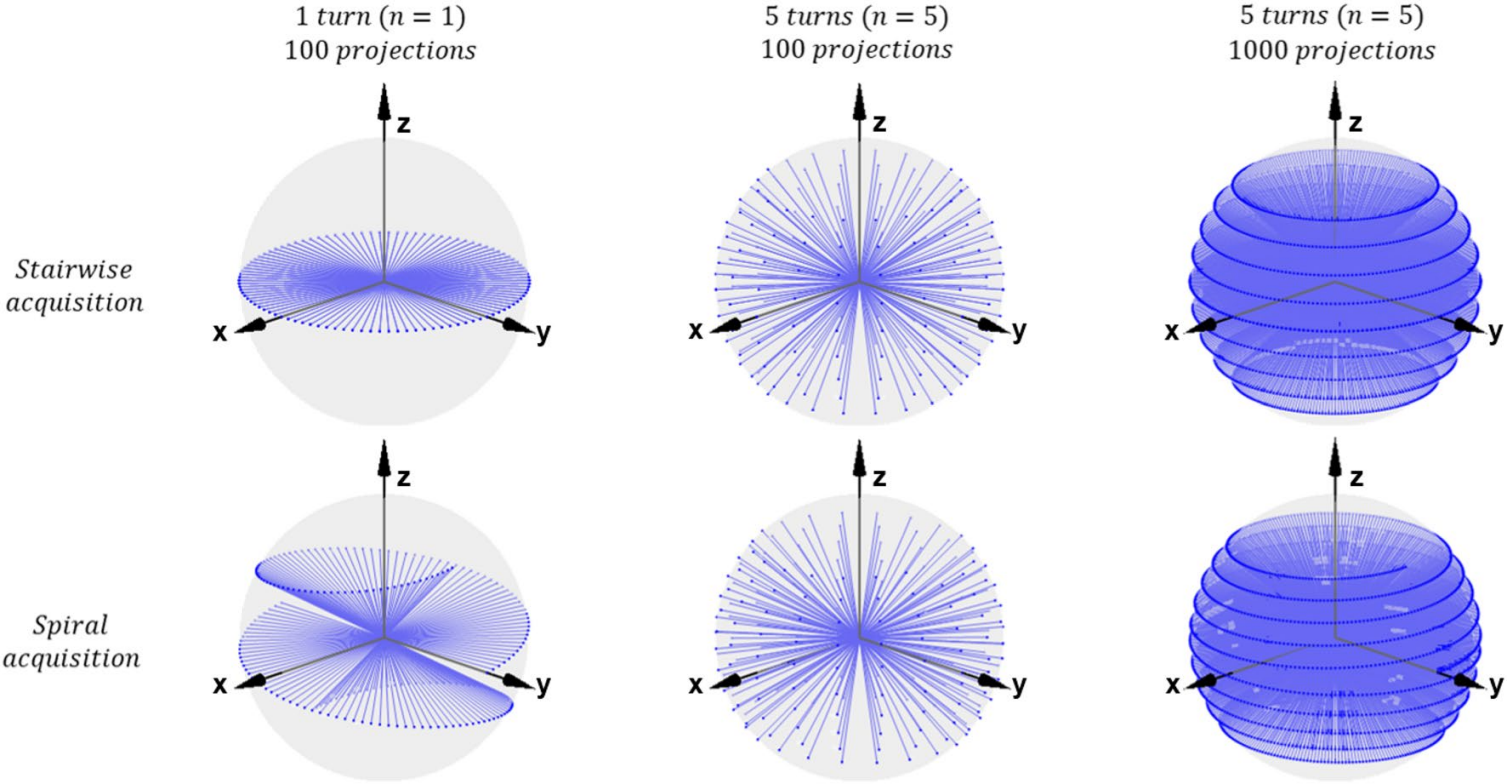
Beam entry/exit trajectories for stairwise and spiral acquisition schemes from the sample’s point of view. The number of turns *n* and the total number of projections are indicated on top of the plots. The tilt angle $\beta$ for the shown stairwise trajectory with $n=1$ is ${0}^{\circ }$ but it can be chosen to be any angle between ${0}^{\circ }$ and ${45}^{\circ }$.

The stairwise acquisition protocols foresee a stepwise tilting of the rotation axis as previously implemented for scanning SAXS, which has omnidirectional scattering sensitivity^17^. The rotation motor turns from $\alpha ={0}^{\circ }$ to $\alpha ={360}^{\circ }$ at each angle $\beta _i$ from the set of chosen *n* tilt angles $\beta =\{\beta _i|\beta _{min}\le \beta _i\le \beta _{max}, i=1, \ldots ,n\}$, resulting in stairwise beam trajectories. The number of projections at each tilt angle is kept constant in this study. In addition to the stairwise acquisition protocol, we also propose a spiral acquisition scheme to push the acquisition speed and minimise overhead. The spiral acquisition trajectory is realised by tilting the rotation axis while the sample is being rotated. The rotation and tilt motors simultaneously turn from $\alpha ={0}^{\circ }$ to $\alpha ={360}^{\circ }\times n$ and $\beta =\beta _{min}$ to $\beta =\beta _{max}$. The spiral acquisition provides projection views at more tilt angles than the stairwise acquisition. The overhead is inherently close to zero for the spiral protocol whereas acquisition overhead in between each tilt angle is difficult to avoid for the stairwise scheme. For both acquisition types, the maximum tilt angle $\beta _{max}$ is limited in our current setup to ${45}^{\circ }$ because the motor stage blocks the beam at larger tilt angles. Beam entry/exit trajectories on a unit sphere from the sample’s point of view for different acquisition protocols are shown in Fig. 2.

The difference in the beam trajectories between the two acquisition types is largest when $n=1$. Acquisitions with $n=5$ and a total of 100 projections show a more uniform distribution of beam entry/exit points over the unit sphere compared to $n=1$ with the same total number of projections. We may expect a higher reconstruction accuracy with $n=5$ compared to with $n=1$. However, for a given total experiment time, the protocol with $n=5$ requires a rotation ($\alpha )$ speed five times higher than for $n=1$. This could lead to increased motion and blurring artefacts especially for time resolved experiments with a short total experiment time; it could therefore be beneficial to minimise *n* in such cases.

### Null space computation

The null space analysis is used to assess a geometric condition for tomographic imaging problems. The existence of a non-trivial null space implies that certain components in the reconstruction volume cannot be confidently recovered from the measurements; therefore, the null space analysis can be used to assess which part of the model can be resolved with a specific acquisition geometry^28^. For example, a null space analysis was used to evaluate the uncertainty of the computed reconstruction for the isotropic scattering component in anisotropic X-ray darkfield tomography (AXDT), with conventional linear gratings^16^.

The null space is the linear subspace of all vectors $\varvec{\mu }$ mapped to zero ($A\varvec{\mu }=\varvec{0}$) and can be derived purely from the system matrix *A* which describes the geometrical setup of an acquisition protocol. A non-trivial null space $\varvec{\mu }_{null}$ always exists when the system matrix *A* is rank deficient. Any vector $\varvec{\mu }$ in such a system can be decomposed into the minimum-norm least square solution $\varvec{\mu }_{LS}$ and the null space $\varvec{\mu }_{null}$ that are orthogonal to each other, holding the following property: $A\varvec{\mu }=A(\varvec{\mu }_{LS}+\varvec{\mu }_{null})=A\varvec{\mu }_{LS}$ because $A\varvec{\mu }_{null}=0$. The computation of the null space based on singular value decomposition (SVD) is efficient for small problems, which requires to load the full representation of *A* into the memory. For larger system, such as our tensor tomography problem, the huge memory requirements of this method prevent using the SVD for the null space computation.

An iterative method is employed here instead: finding the null space is equivalent to solving the following constrained optimisation problem:

1 $$\begin{aligned} \min _{\varvec{\mu }_{null}}\frac{1}{2}{\Vert \varvec{\mu }_{null} - \varvec{v}\Vert }_2^2&\text {subject to}&A\varvec{\mu }_{null}=\varvec{0}. \end{aligned}$$

$\varvec{v}$ is a random vector whose elements are in the range of [0, 1]. Solving the above problem is equivalent to computing the orthogonal projection of the initial vector $\varvec{\mu }_{null}^0$ on the null space $\mathscr {N}(A)$. An approximate solution $\varvec{\mu }_{null}$ is found by setting the gradient of the Lagrange function $\mathscr {L}(\varvec{\mu }_{null}, \varvec{\lambda })$ of the above problem to zero and iteratively solving it, where $\varvec{\lambda }$ is the Lagrange multiplier vector.

2 $$\begin{aligned} \nabla \mathscr {L}(\varvec{\mu }_{null}, \varvec{\lambda })=\varvec{0}\Leftrightarrow & {} \begin{bmatrix} I &{} A^T\\ A &{} 0 \end{bmatrix} \begin{bmatrix} \varvec{\mu }_{null}\\ \varvec{\lambda } \end{bmatrix} = \begin{bmatrix} \varvec{v}\\ \varvec{0} \end{bmatrix} \end{aligned}$$

### Assessment of the reconstruction accuracy

To assess and quantify the reconstruction results in scattering tensor tomography, more aspects than in conventional scalar tomography need to be taken into account because a single voxel is characterised by more than one parameter. Analogously to scalar tomography, the distance between the tensor in each voxel for the reference and the evaluated reconstructed volumes can be computed. We use here the inner product of the structure main orientation vectors $\varvec{v}_{ref}$ and $\varvec{v}$, $I = |\langle \varvec{v}_{ref}, \varvec{v}\rangle |$ in each voxel, as the error metric to assess the orientation reconstruction accuracy of data acquired with different acquisition protocols, where $\varvec{v}_{ref}$ and $\varvec{v}$ are the reconstructed structure orientation vector of the reference and the assessed volume respectively. In addition to the error in the reconstructed orientation, we also use an error metric $E = ({s_{ref} - s})^2$ to compare the average scattering or scattering anisotropy signal *s*, which is a scalar variable, extracted from the reconstructed tensor in each voxel.

## Results

### Null space analysis

**Figure 3:**
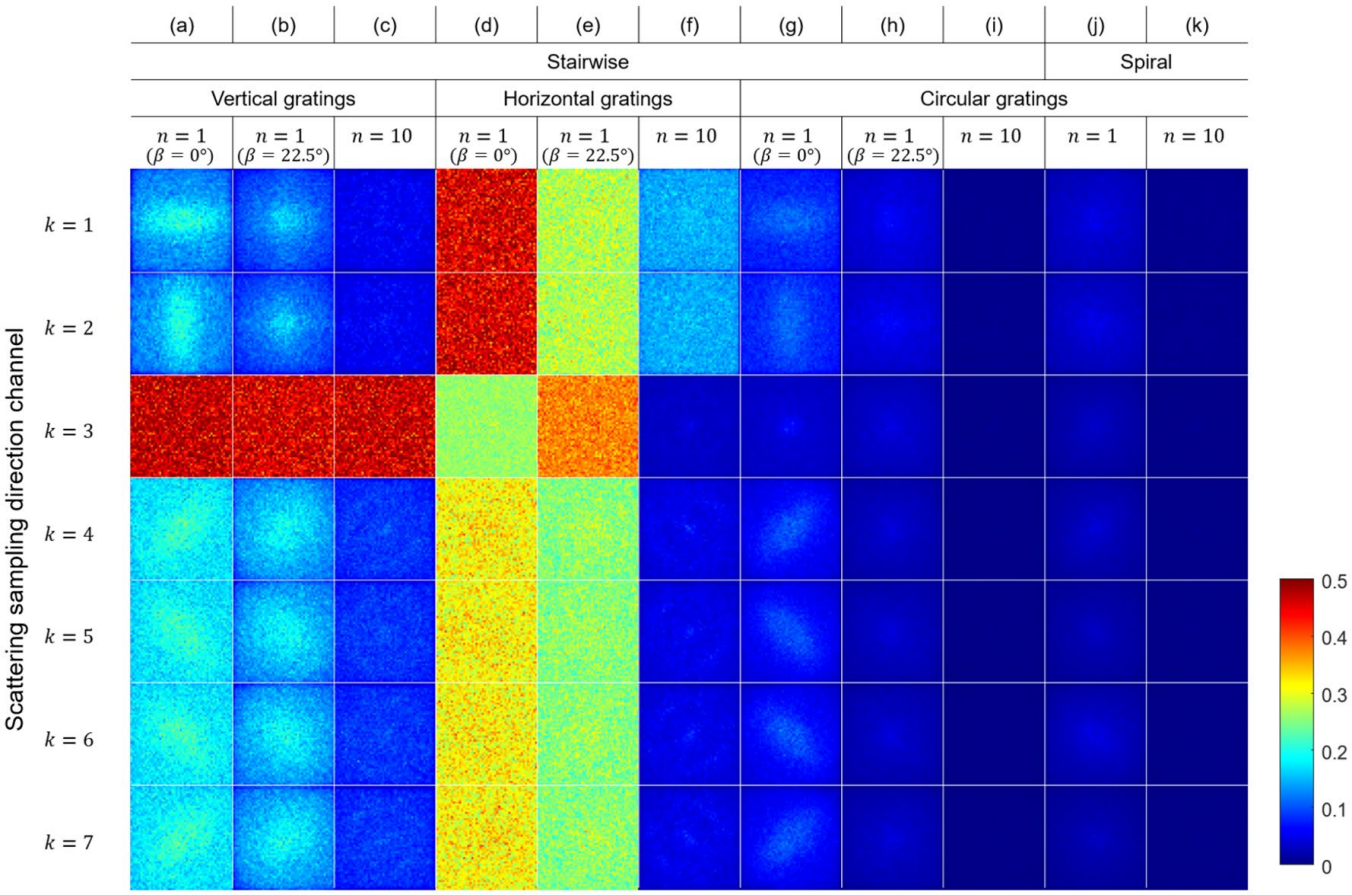
Visualisation of the computed null space for different scattering sampling directions *k*. The null space was computed for different acquisition geometries: stairwise and spiral acquisition; different grating types: vertically aligned linear gratings, horizontally aligned linear gratings, and circular gratings; different number of turns *n*. $\beta$ indicates the tilt angle. High values in null space for scattering component *k* indicate that the acquisition protocol provides large uncertainties in the reconstruction for that component.

The null space was computed for different system matrices for different acquisition protocols for an example reconstruction volume size of $50\times 50\times 50$ voxels. The total number of projections was fixed to 1000 for all acquisition protocols, and the number of unit cells per projection was $100\times 100$ The computed null space was averaged along the z-direction (parallel to the rotation axis when the tilt angle is zero) for different scattering sampling directions and visualised in Fig. 3. The scattering sampling directions should not be confused with the structure orientation. High values in the null space indicate that the acquisition protocol provides large uncertainties in the reconstruction results. Considering that the elements of the initial random vector $\varvec{v}$ are in the range of [0, 1], the highest value would be 1. Considering that the elements of the initial random vector $\varvec{v}$ are in the range of [0, 1] and that difference between $\varvec{v}$ and $\varvec{\mu }_{null}$ is minimised (Eq. 1), the elements of $\varvec{\mu }_{null}$ should also be in this range, with the highest values around 1. Figure 3a–e show that the information for certain scattering sampling directions cannot be reconstructed with the conventional linear grating arrays, independently on whether the linear grating array is aligned vertically ($\gamma ={0}^{\circ }$) or horizontally ($\gamma ={90}^{\circ }$). The null space is reduced when $n=10$ and the linear grating array is aligned horizontally as shown in Fig. 3f, even though the null space for $k=1,2$ remains higher compared to the other components. On the other hand, a significantly larger reduction in the null space is achieved by using a circular grating array already when $n=1$ ($\beta ={0}^{\circ }$) as shown in Fig. 3g. Choosing a tilt angle of $\beta ={22.5}^{\circ }$ instead of $\beta ={0}^{\circ }$ or using the spiral acquisition protocol instead of the stairwise one gives a smaller null space as shown in Fig. 3h,j, respectively. We observe a general reduction in the null space when *n* is increased from 1 to 10 both for the stairwise ($\beta =\{{0}^{\circ },{5}^{\circ }, \ldots ,{45}^{\circ }\}$) and spiral ($\beta _{min}={0}^{\circ }$ and $\beta _{max}={45}^{\circ }$) acquisition geometry. Little difference is observed between the stairwise and spiral acquisition protocols when $n=10$ as we can see in Fig. 3i,k. Summarizing, an improvement in the tensor reconstruction accuracy with a circular grating array even at small *n* compared to the case of linear grating arrays is expected. Also, when $n=1$, a higher reconstruction accuracy with the spiral acquisition protocol should be achieved compared to results with a fixed tilt angle of $\beta ={0}^{\circ }$. Instead, comparable results are expected for the spiral acquisition ($n=1$) and for a fixed tilt angle of $\beta ={22.5}^{\circ }$. The null space for $n=2$ and $n=5$ are available online in the Supplementary Fig. S1.

### Simulation study

**Figure 4:**
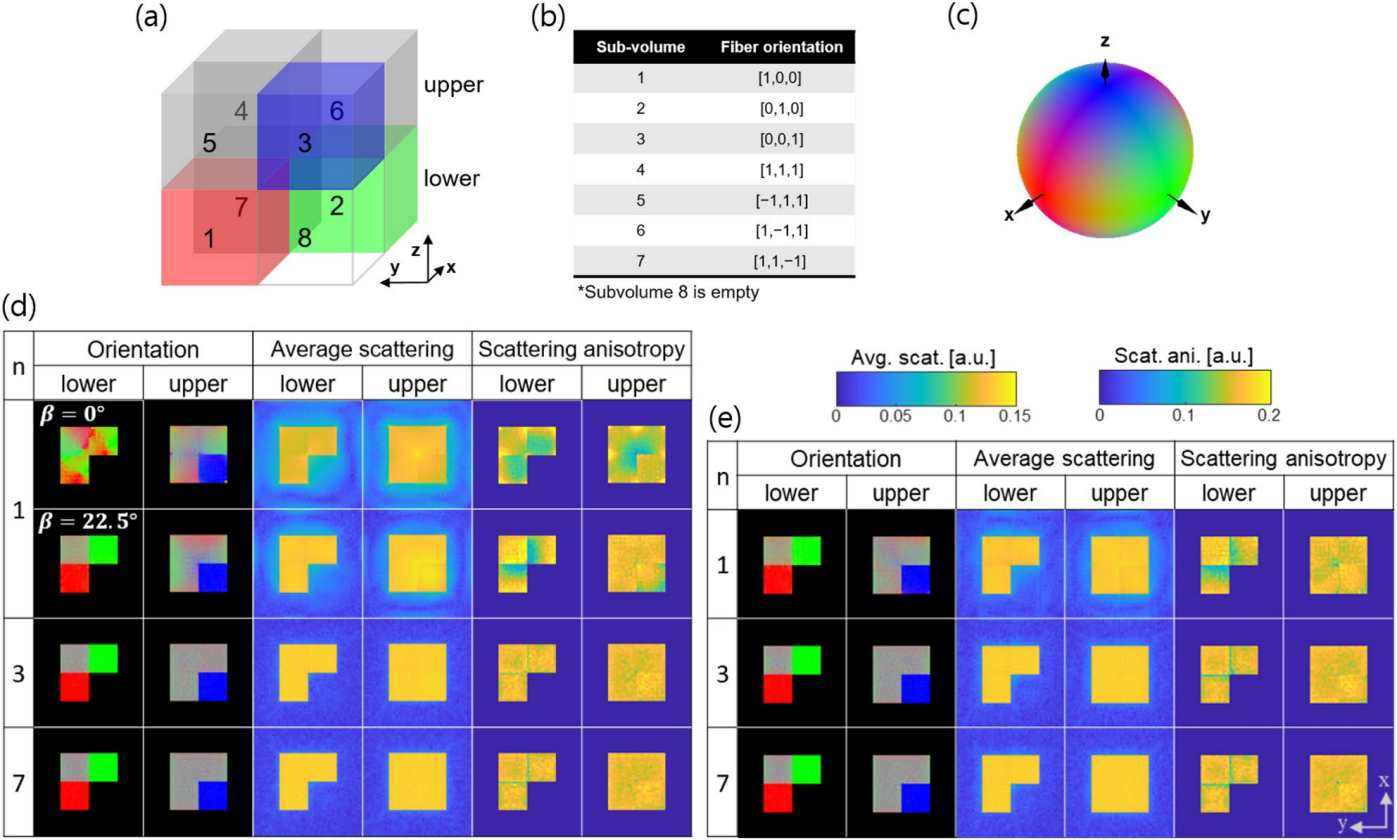
Visualisation of the reconstructed volumes for the in silico sample (**a**), which has different subvolumes with fibres oriented in different directions $o_{fibre}$ (**b**). The orientation vector is visualised according to a colour scheme (**c**) which maps the absolute value of its components [x,y,z] to the [R,G,B] values. Axial slices through the lower and upper part of the reconstructed sample acquired with the stairwise (**d**) and spiral (**e**) acquisition protocols are shown. *n* represents the number of turns. The colour bars for the average scattering and scattering anisotropy are shown on the right.

In a first step we have assessed the performance of the proposed acquisition protocols by conducting experiments with an in silico sample shown in Fig. 4a. The cubic sample is divided into eight equal subvolumes with fibres oriented in different directions as described in Fig. 4b. The fibre orientation in each voxel is colour coded with the [R,G,B] values corresponding to the absolute values of the components [x,y,z] of the vector representing the fibre direction. The corresponding colour mapping sphere is shown in Fig. 4c. This colour coding scheme for the visualisation of 3D orientations is used throughout this paper. The reference volume $\varvec{\mu }_{k,ref}$ was generated by calculating the squared inner product of the scattering sampling directions and the fibre orientation $|\langle \varvec{S}_k, \varvec{o}_{fibre}\rangle |^2$. System matrices for different acquisition geometries were computed. Projection data were generated by forward projection of the reference volume using these system matrices rather than by simulating individual fibres and using a wave propagation approach. This is because we focus on evaluating the effect of different number of tilt angles on the reconstruction accuracy rather than trying to more precisely mimic the small-angle scattering phenomenon of the X-rays in the sample. Additive zero-mean white Gaussian noise proportional to the absorption signal was added to the simulated projections. This is a valid approximation assuming a high-flux environment^29^.

**Figure 5:**
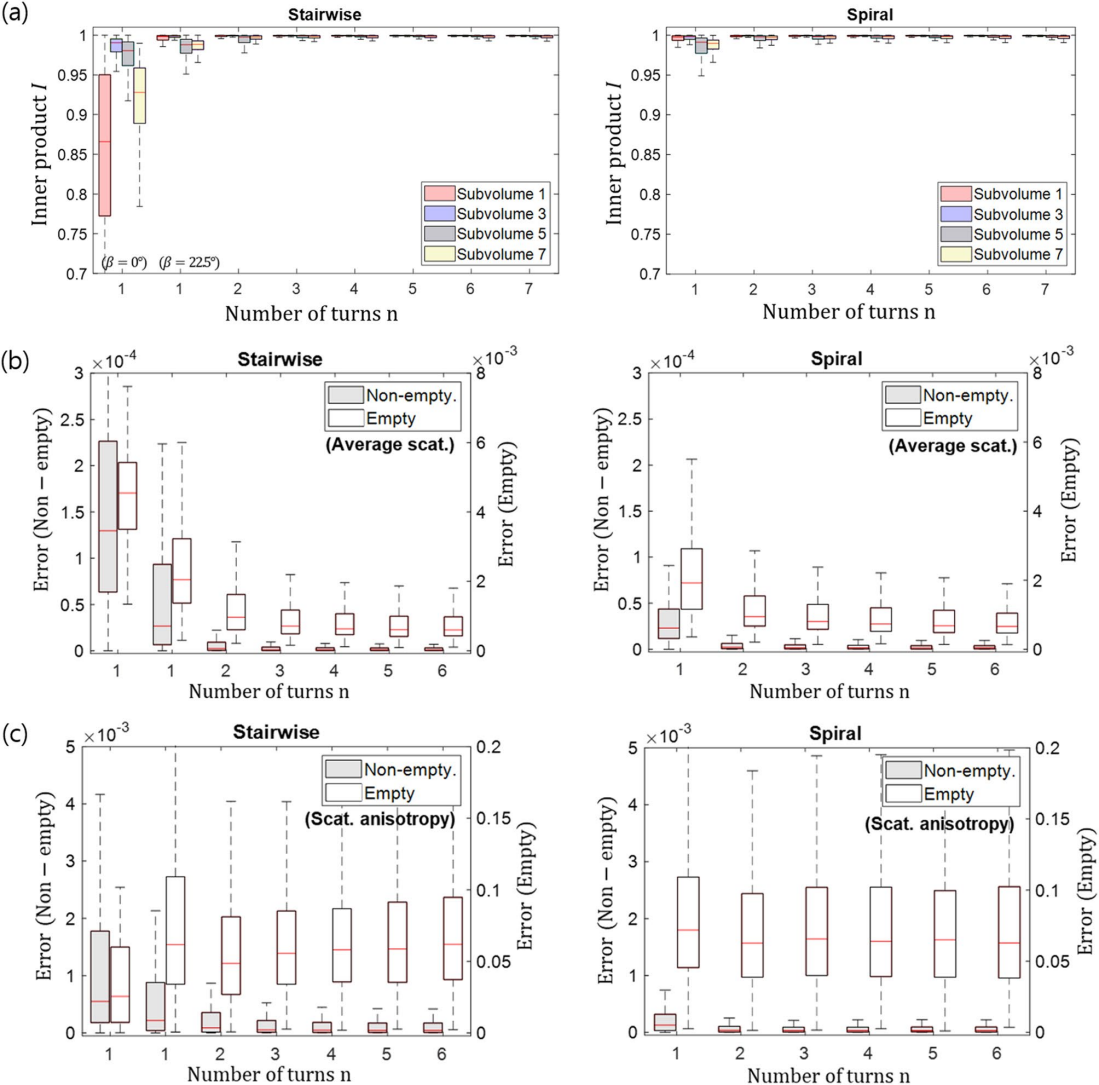
Quantitative assessment of the reconstruction accuracy for the in silico sample. Box plots of the inner product *I* for the different preferential orientations are shown in (**a**). The error *E* of the average scattering is shown in (**b**) and of the scattering anisotropy in (**c**). Note the separate axis scales of the non-empty and the empty volume.

Projection images were computed for different angular positions with *n* from 1 to 7 both for the stairwise and the spiral acquisition geometries. The total number of projections was fixed to 1000, and the number of unit cells per projection image was $100\times 100$. Axial slices through the reconstructed volumes are shown in Fig. 4d,e for $n=1,3,7$. Ideally in this simulation study, the average scattering and scattering anisotropy should have the same value in every voxel for subvolumes 1 to 7. As predicted from the null space analysis, the largest deviations from the reference volume are observed for $n=1$ with a fixed tilt angle of $\beta ={0}^{\circ }$. The reconstruction for $\beta ={22.5}^{\circ }$, $n=1$ and the stairwise acquisition looks closer to the reference than the $\beta ={0}^{\circ }$ case. Reconstructions for all *n* and the spiral acquisition protocol look instead fairly similar, with larger variations in the average scattering and scattering anisotropy. For $n \ge 3$, the reconstruction results for the stairwise and spiral acquisition geometries are comparable. For a more quantitative assessment of the reconstruction results, the inner product *I* of the reconstructed orientation vectors with the ground truth reference vectors was calculated for all voxels of the different subvolumes and the results are shown in the form of box plots in Fig. 5a.

For the stairwise acquisition protocol, the largest inaccuracy is observed for $n=1$ for subvolume 1 and 2 where the fibres lie in the horizontal (x–y) plane as could already be visually appreciated in Fig. 4d. The subvolume 3 is relatively well reproduced even for $n=1$ as expected because we have more projection poses where the X-ray beam is orthogonal to the fibre structure. In Fig. 5a, we observed a larger orientation inaccuracy when $n=1$ for subvolume 7 with diagonally oriented fibres compared to other subvolumes with diagonally oriented fibres. This observation arises from the fact that the directional scattering information in subvolume 7 is contaminated by signals from the neighbor subvolumes 1 and 2, where the fibres aligned in the xy-plane scatter isotropically in a certain angular range of projection. It means that the reconstruction accuracy depends not only on the acquisition scheme but also on the surrounding structures around the region of interest. The inner product *I* approaches 1 when $n\ge 3$ for all subvolumes.

For the spiral acquisition geometry, only minor variations in the inner product *I* for different *n* and subvolumes exist. For the error *E* analysis of the average scattering and scattering anisotropy results (Fig. 5b,c), the subvolumes were grouped into non-empty (1–7) and empty (8). In general, the error *E* for both the average scattering and scattering anisotropy signal was larger in the empty subvolume, but no significant difference in error *E* is observed for different *n* and geometries except for $n=1$ and the stairwise acquisition protocol. In addition, the inner product *I* and the error *E* were similar for a fixed tilt angled of $\beta ={22.5}^{\circ }$ and the spiral acquisition when $n=1$ as predicted by the null space analysis (Fig. 3h,j). The results shown here are obtained with 1000 projections, which for the considered volume size of $50\times 50\times 50$ guarantee sufficient sampling. Even with this largest number of projections, a fixed tilt angle at $\beta ={0}^{\circ }$will not provide accurate results. The same analysis has also been performed with only 100 projections (sparse sampling) with the results following the same trend regarding *n* and the accuracy metrics as reported from 1000 projections.

### Validation sample

**Figure 6:**
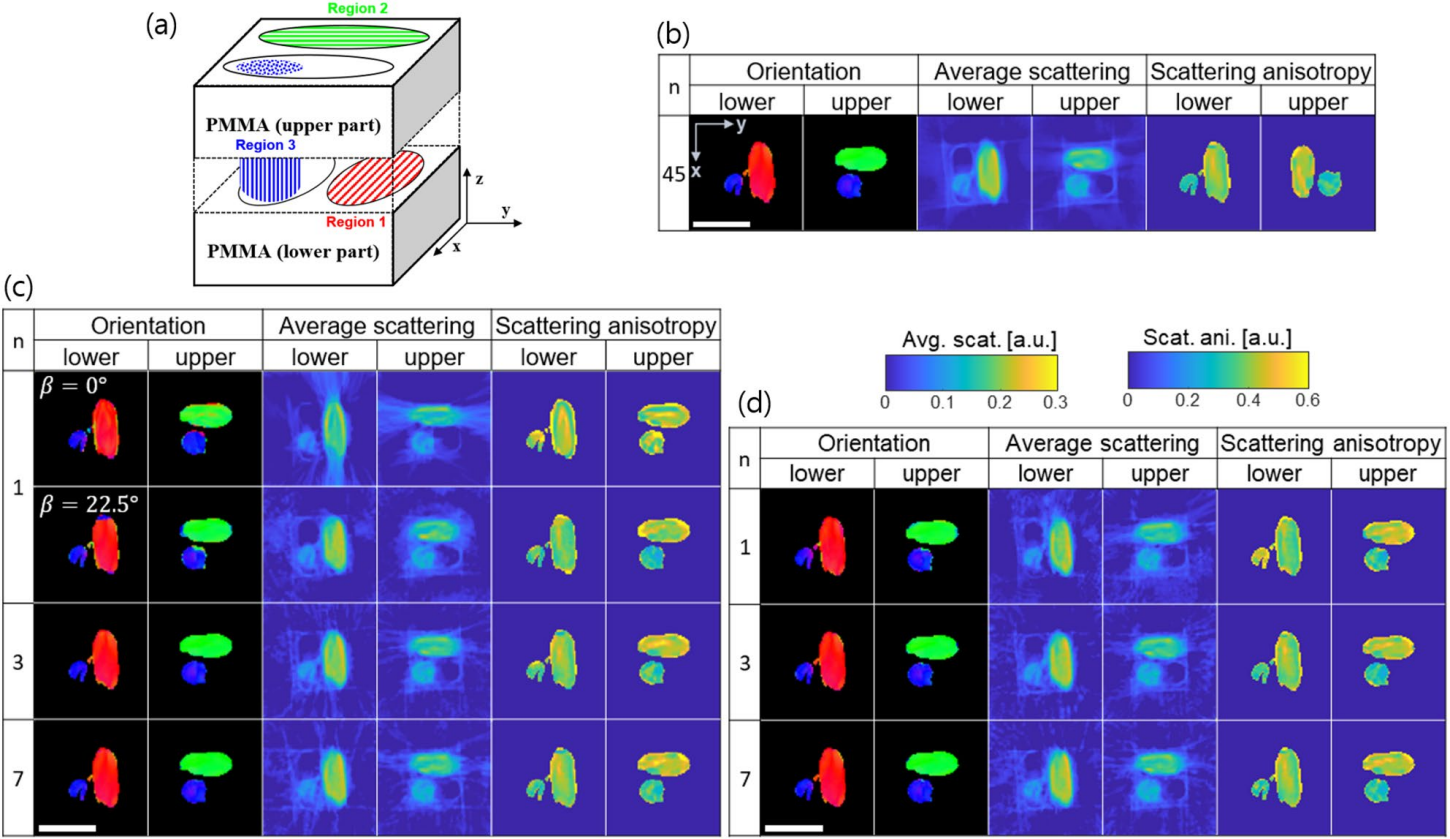
Visualisation of the reconstructed volumes for the validation sample. The validation sample (**a**) with three carbon fibre bundles in a polymethyl methacrylate (PMMA) box oriented orthogonal to one another. Axial slices through the lower and the upper part of the validation sample reconstructed from the data acquired with the reference (**b**), the stairwise (**c**) and the spiral (**d**) acquisition protocols. Fibre orientation, average scattering and scattering anisotropy values are shown for each acquisition scheme. Scale bar: 4 mm

A validation sample (Fig. 6a) with a size of $4\times 4\times 4\mathrm{mm}^{3}$ as described in our previous publication^21^ was used. The sample has carbon fibres with a diameter of $12\ \upmu \mathrm{m}$ bundled and inserted into three different regions indicated as 1, 2, and 3. The projection images were acquired with a parallel synchrotron monochromatic X-ray beam ($17 \mathrm{keV}$, bandwidth of 2–3$\%$) at the TOMCAT beamline, Swiss Light Source, Paul Scherrer Institut. The beam size was $14.3\times 4.8\;\mathrm{mm}^2$ and the effective FOV achieved with vertically stitched scans was $14.3\times 12.7\;\mathrm{mm}^2$. The $\pi$-shift circular grating array had a unit cell period of $P=49.5\ {\upmu }\mathrm{m}$ and a fine grating period of $g=1.46\ {\upmu }\mathrm{m}$ (Fig. 1b). The number of unit cells (circular patterns) in the FOV was $144\times 128$. A LuAG:Ce scintillator with a thickness of ${300}\;{\upmu }\mathrm{m}$ was used to convert incoming X-rays to photons in the optical energy range compatible with the used camera and was placed at a distance $L=49.5\;\mathrm{cm}$ (Fig. 1a) from the grating array. Visible light photons were delivered to the detector by a high numerical aperture 1x microscope optics accepting a diagonal up to 40 mm. The in-house developed GigaFRoST^30^ sCMOS detector with a sensor pixel size of ${11}\;{\upmu }\mathrm{m}$ was used. The fringe pattern in each unit cell was sampled with $9\times 9$ detector pixel windows. The exposure time for each projection image was $10\ \mathrm{ms}$ and projection images were acquired in a continuous manner.

The reference reconstruction visualised in Fig. 6b was generated with the stairwise acquisition scheme with projection images taken at 100 rotation and 46 tilt angles: $\alpha =\{0^{\circ },3.6^{\circ }, \ldots , 356.4^{\circ }\}$ and $\beta =\{0^{\circ },1^{\circ }, \ldots , 45^{\circ }\}$. The reconstructed volume was used as reference (“ground-truth”) for the qualitative and quantitative comparison of the different acquisition protocols. For the results for the stairwise acquisition scheme shown in Fig. 6c, the full reference dataset was uniformly sub sampled for the desired *n* values so that always a total of 100 projection images was used for the reconstruction. For instance, 100 projection images were taken at tilt angle $\beta =0^{\circ }$ for $n=1$, and 33 projection images were taken at each tilt angle $\beta =\{0^{\circ },23^{\circ },45^{\circ }\}$ for $n=3$. For the spiral acquisition geometry, a total of 100 projection images homogeneously distributed along the spiral trajectories with different *n* values, where $\beta _{min}=0^{\circ }$ and $\beta _{max}=45^{\circ }$, were acquired and considered for the reconstruction (Fig. 6d). A stitched scan was performed due to the limited beam height and the sample translation for three stitches caused a few seconds of overhead. For the different geometries, the tensor reconstruction was performed following the method described in Ref.2.1, with a volume geometry of $81\times 81\times 76$ voxels.

**Figure 7:**
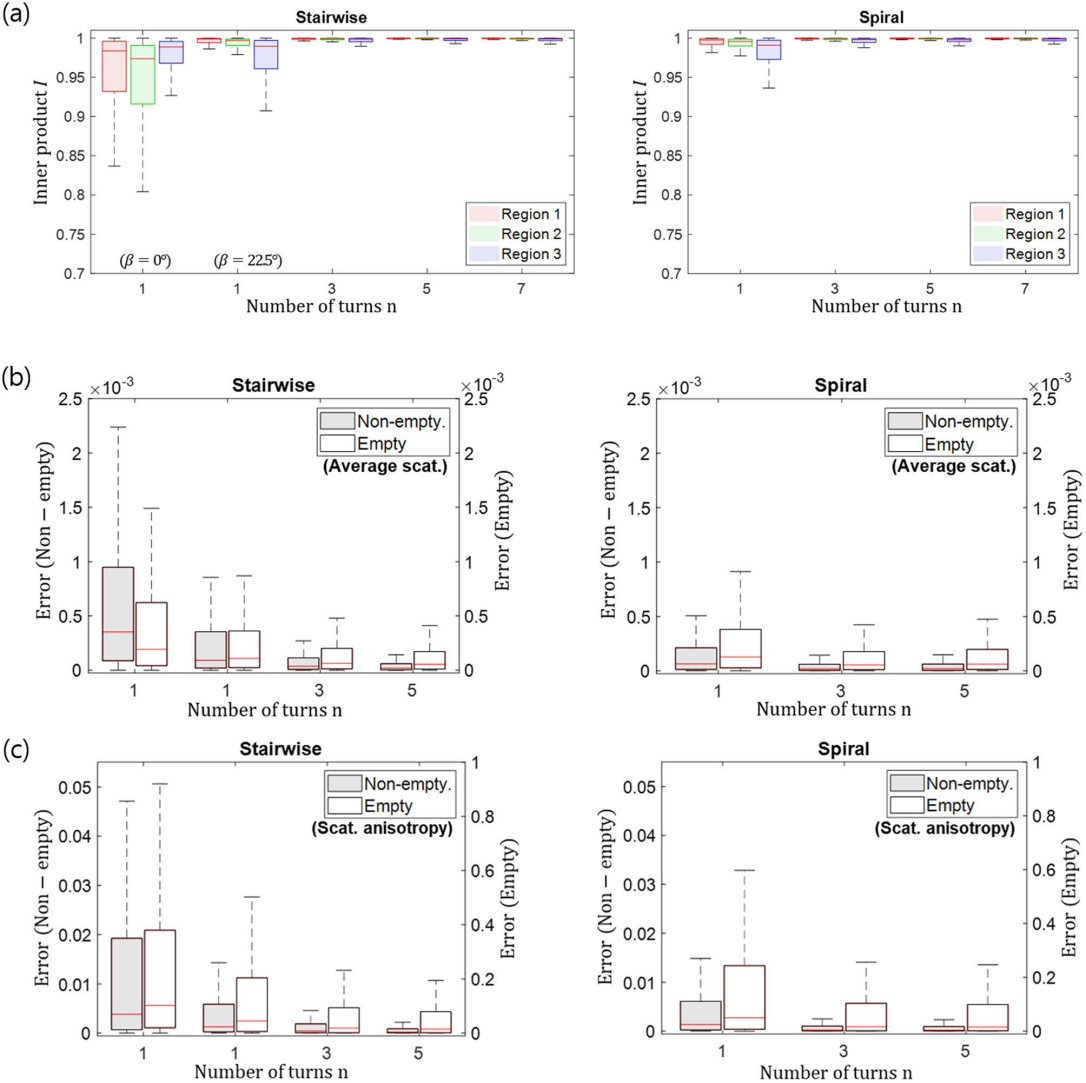
Quantitative assessment of the reconstruction accuracy for the validation sample. Box plots of the inner product *I* for the different preferential orientations are shown in (**a**). The error *E* of the average scattering is shown in (**b**) and of the scattering anisotropy in (**c**). Note the separate axis scales of the non-empty and the empty volume.

From the axial slices shown in Fig. 6b–d, it is clear that the reconstruction with a fixed tilt angle of $\beta =0^{\circ }$ deviates the most from the reference images. The reconstructions improved for $\beta =22.5^{\circ }$ or for spiral acquisition when $n=1$ although they still visually deviate from the reference. Only minor deviation from the reference is instead visually observable for all other cases. These observations are qualitatively in agreement with the null space analysis and the simulation results shown in the previous sections. In addition to the visual comparison of the reconstruction results, we have also performed a quantitative analysis with the inner product *I* and the scalar error *E*. For the results evaluation (Fig. 7), voxels belonging to the 3 different regions shown in Fig. 6a have been considered. The inner product *I* was calculated for each region defined as the group of voxels in a box surrounding the region and the results are shown in Fig. 7a. We observe relatively more deviation from the reference with a fixed tilt angle of $\beta =0^{\circ }$ compared to the other cases. Also, the deviation is larger for the region 1 and 2 where the fibres lie in the horizontal (x–y) plane. As projection views at tilt angles larger than $0^{\circ }$ are added, the reconstruction accuracy in the x–y plane increases. For $n\ge 3$, the reconstructed orientation is already close to the reference for both the stairwise and spiral acquisition schemes. For the calculation of the error *E*, the reconstructed volume was subdivided in empty and non-empty regions by applying a threshold to the average scattering. The group of voxels with an average scattering signal higher than 30$\%$ of the maximum signal over the entire reference volume was defined as the non-empty region and the group of voxels with a signal smaller than the defined threshold was defined as the empty region. For the pixels in both regions, the error *E* was calculated and the box plots are shown in Fig. 7b,c. In general, the error *E* was largest when $n=1$ for the stairwise acquisition, whereas no major difference is observed for $n\ge 3$, in agreement with the analyses in the previous sections. The error *E* for the scattering anisotropy in the empty regions was particularly large (Fig. 7c) as also observed in the simulation study (Fig. 5c). We believe that this is because the scattering anisotropy is proportional to the square root of the ratio of the variance to mean of the lengths of the principal axes of the scattering ellipsoid and is inherently more prone to noise. In particular in background regions where the average scattering contribution is small or none and at sharp edges where the scattering direction changes over a short distance, scattering anisotropy signals could appear artificially large. A mask based on either the average scattering or the absorption is therefore necessary to properly visualise volumes with the scattering anisotropy. The simplest type of such a mask sets all voxels with a value below a certain threshold to zero. A threshold based mask was created from the average scattering information and applied to the scattering anisotropy visualisation in Fig. 6b–d. For the scattering anisotropy visualisation in the simulation study (Fig. 4d,e), all voxels outside the ground truth sample boundary were set to zero.

### Fibre pellet assembly phantom

**Figure 8:**
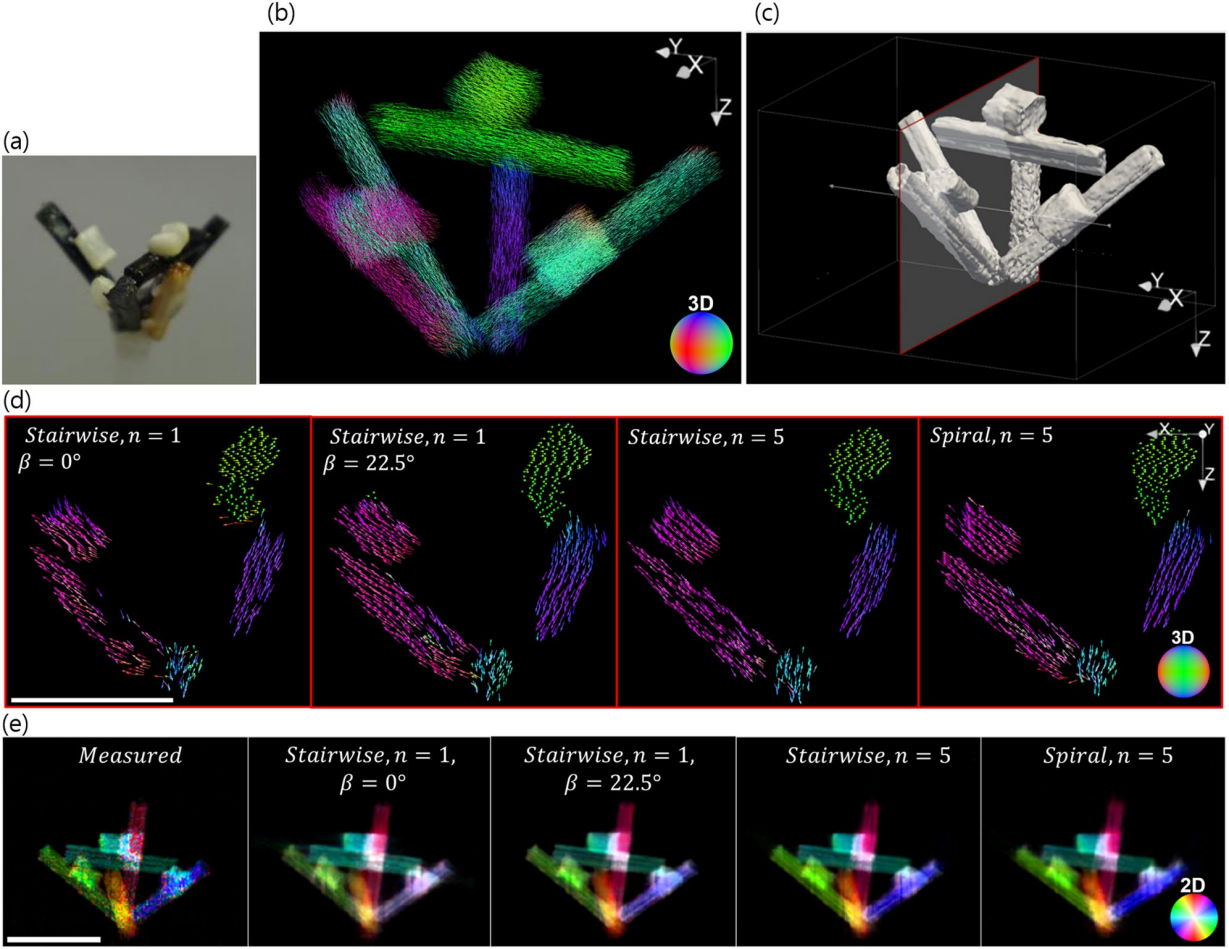
Fibre pellet phantom analysis—(**a**) The sample is composed of carbon (black) and glass fibre (white) material as well as wood (brown). (**b**) Full 3D view of the reconstructed fibre orientations (stairwise, $n=5$). (**c**) Contour image based on the average scattering signal. (**d**) Reconstructed fibre orientation for the 2D slice shown in (**c**) (subsampled by a factor of 5 for visualisation). The 3D colour ball maps the 3D fibre orientation. (**e**) Measured and forward projection images at an arbitrary angle. The 2D colour wheel maps the fibre orientations projected onto the 2D detector plane. Scale bar: 1 cm.

The fibre pellet phantom shown in Fig. 8 which is an assembly of different stick pellets composed of industrially relevant fibre-reinforced materials was measured and analysed. This assembly is a multi-material validation sample where the fibre or tubular structures are aligned along the axial direction of each stick as can be seen in the Supplementary Fig. S2. The experimental conditions were the same as in “Validation sample” except that the projection images were acquired with $30\ \mathrm{keV}$. The used high-aspect ratio circular grating array specific for $30\ \mathrm{keV}$ was fabricated with an optimised deep reactive ion etching process into a silicon substrate with a circular pattern mask generated by e-beam lithography^24^. This $\pi$-shift circular grating array had the same dimension as the array used in the previous experiment but a different grating depth dictated by the chosen energy. The beam size was $11.6\times 3.5\;\mathrm{mm}^2$ and the effective FOV achieved with stitched scans was $26.2\times 22.5\;\mathrm{mm}^2$. The number of unit cells (circular patterns) in the FOV was $264\times 227$. The scintillator-detector assembly was placed at a distance $L=74.1\ \mathrm{cm}$ from the grating array. One hundred projection images were acquired in a continuous manner and the exposure time for each projection image was $10\ \mathrm{ms}$.

The sample was reconstructed from data acquired with the stairwise acquisition scheme with $n=1$ and $n=5$. It was also reconstructed from data acquired with a spiral acquisition protocol with $n=5$. With all acquisition schemes used, it was possible to recover, at least qualitatively, the expected fibre orientations as shown in Fig. 8d. In order to provide a comparison with the measured data, an arbitrary projection angle was chosen at a tilted view of $22.5^{\circ }$. The measured and the forward projection images for this angle are shown in Fig. 8e. The directional scattering signal (visibility reduction) *V* in the projection plane was modelled with a cosine function $V(\gamma )=a_0+a_1\cos {(\gamma -\gamma _{main})}$^20^. The three parameters $a_0$, $a_1$ and $\gamma _{main}$ were extracted by Fourier analysis. $a_0$ represents the average scattering and the degree of anisotropy is computed as the ratio $a_1/a_0$. $\gamma _{main}$ is the main direction of the underlying structure. These three parameters are coded with the HSV colouring scheme: H as the main orientation, S as the degree of scattering anisotropy, V as the average scattering. The colour coded projection images are shown in Fig. 8e where H and S are mapped by the colour wheel. V appears as the brightness of the pixel (dark as V approaches 0, bright as V approaches 1). The acquisition protocol with $n=1$ ($\beta =0^{\circ }$) gave a forward projection image which deviated the most from the measured projection whereas the other two protocols with $n=5$ gave much similar projection images. These experimental results are in agreement with the previous sections.

## Discussion

The in-silico as well as the experimental studies show that simple protocols compatible with fast acquisition schemes are possible. In general for higher accuracy the number of turns *n* has to be at least $> 1$. If the acquisition speed needs though to be maximised, for instance for studying fast dynamic processes, the spiral acquisition protocol with $n=1$ can provide sufficient accuracy for many applications. If even further simplification is unavoidable, for example if using complex sample environments for which fast simultaneous rotation around 2 axes is cumbersome, acquisition at a fixed tilt angle is also a viable solution. This fixed tilt angle should though be chosen carefully and in most cases the trivial selection of $\beta =0^{\circ }$ is not the optimal choice as shown by the null space analysis and the different reconstruction studies. This is because of the missing wedge problem for a certain angular range where the fibres are aligned parallel to the beam and all fibres on the axial plane are affected by the missing wedge problem with a fixed tilt angle of $\beta =0^{\circ }$. A fixed tilt angle of $\beta =22.5^{\circ }$ provides superior results compared to the trivial choice and similar accuracy to the spiral protocol with $n=1$ because of beam vectors off the axial plane unlike the case of a fixed tilt angle of $\beta =0^{\circ }$. Even though a system with 2 axes of rotation is in principle required also for the fixed tilt angle case to ensure flexibility in the selection of $\beta$, rotation around a single axis during data acquisition strongly simplifies all synchronisation aspects, not irrelevant at high speed, potentially leading to less uncertainties in the reconstruction process of the final volume. For specific applications, e.g. to increase the stability of the setup, a single rotation stage firmly mounted with the rotation axis at a defined angle with respect to the beam can also be an option to consider.

The advantage of *n* at least $>1$ by a constant number of projection is clear and a relevant observation was made by a previous scanning SAXS based tensor tomography study that the reconstruction accuracy is more related to having a sufficient number of turns *n* than having a larger total number of projections^17^. For the reconstruction volume sizes in our experimental studies, 100 projections lead to undersampled tomographic datasets; we have nonetheless decided to use such a small number of projections with small *n* values in the presented experiments to effectively push the acquisition speed with fast tensor tomography applications in mind. The effect of further reducing the number of projections leading to extremely sparse datasets in conjunction with the proposed acquisition protocols needs though to be investigated but is beyond the scope of this study.

A direct quantitative comparison of our method to existing tensor tomography methods of all involved aspects is not straightforward and beyond the scope of this work. Our method in general has a larger voxel size compared to the existing methods and thus it may yield lower spatial resolution with the absorption contrast. However, microstructure is indirectly revealed by the small-angle scattering signal in scattering tensor tomography rather than directly resolved by detector pixels. Thus, spatial resolution is often not of the main importance for tensor tomography and the effective spatial resolution is rarely reported in published studies. Scans are rather optimised for different purposes and different samples, and different sources are used. We still would like to note that it is in general clear that the large acquisition overhead intrinsic in the scanning SAXS geometry limits this method to small samples and makes it unsuitable for imaging industrially relevant large volumes or dynamic processes. On the other hand, reported grating interferometric studies with linear gratings are almost exclusively performed on conventional lab-based sources. Therefore, it is meaningless to directly compare the scan time because the exposure time for a single radiographic projection is significantly longer than at a synchrotron. Nevertheless, the need of rather complex trajectories and phase stepping with linear gratings causes frequent discontinuities in the acquisition process increasing the overall overhead even if this technique would be used with a higher flux source.

With our method at synchrotron facilities, the exposure time for each single radiographic projection is usually in the order of a few to a few tens ms. Using an $n=1$ protocol with 100 projections continuously acquired, mostly sufficient for the experimental configurations used in this study, one full omni-directional scattering tensor tomographic volume covering e.g. $16\times 16\times 4\ \mathrm{mm}^{3}=1 \ \mathrm{cm}^3$, with a spatial resolution in the order of 50 to $100 \; \upmu \mathrm{m}$ can be acquired in less than $1 \; \mathrm{s}$. This means that macroscopic, statistically relevant samples (e.g. ${10}\ \mathrm{cm}^3$) can be routinely investigated in a matter of minutes even taking into account the overhead related to sample movements for stitched scan. Alternatively, if a dynamic process is of interest, it is possible to follow the same evolving volume through time.

## Conclusion

In this study, comparative analyses were presented for simple acquisition protocols optimally suited for rapid scattering tensor tomography. First, the null space was significantly reduced by substituting conventional linear with circular gratings already when the tilt angles or the number of spiral turns *n* were just 1. This first mathematical evidence highlighted the potential of using a circular grating array for fast scattering tensor tomography with simple acquisition geometries with a fairly small *n*. Second, simulation studies with an in silico sample showed that the reconstructed volumes were already close to the ground truth with $n\le 3$, regardless of the used acquisition protocol. Lastly, experimental studies provided results that were in agreement with the null space analysis as well as with the simulation results demonstrating the robustness of the technique with respect to the unavoidable experimental uncertainties.

We conclude that, with a circular grating array, the suggested simple acquisition schemes with a fairly small number of turns *n* are inherently sufficient in providing directional scattering information for scattering tensor tomography. Thus, the proposed acquisition protocols build the basis towards rapid inspection and/or time-resolved X-ray scattering tensor tomography in the near future. In principle, the proposed acquisition protocols are compatible with and can be easily transferable to X-ray tube setups and the range of applications can be expanded to industrially more relevant problems. In addition, the presented framework of the null space analysis will be useful to assess and optimise in advance an arbitrary acquisition protocol for X-ray scattering tensor tomography. Even though this work is based on a circular grating array, the proposed acquisition protocols can actually to be also directly transferred to any tensor tomography modality with omnidirectional scattering sensitivity such as speckle pattern and omnidirectional simultaneous reciprocal and real space imaging based methods.

## Supplementary Information

Supplementary Information.

## Data Availability

The datasets generated and analysed during the current study are available from the corresponding author on reasonable request.
