# Author Correction: Fast acquisition protocol for X-ray scattering tensor tomography

**DOI:** 10.1038/s41598-022-05324-6

**Published:** 2022-01-24

**Authors:** Jisoo Kim, Matias Kagias, Federica Marone, Zhitian Shi, Marco Stampanoni

**Affiliations:** 1 Institute for Biomedical Engineering, University and ETH Zürich, 8092 Zurich, Switzerland; 2 Swiss Light Source, Paul Scherrer Institut, 5232 Villigen, Switzerland; 3 Present Address: Division of Engineering and Applied Science, California Institute of Technology, Pasadena, CA 91125 USA

Correction to: *Scientific Reports* 10.1038/s41598-021-02467-w, published online 29 November 2021

The original version of this Article contained errors in Figures 5 and 7 where the graphs in panels (a), (b) and (c) were incorrectly ordered. The original Figures 5 and 7 and accompanying legends appear below.

**Figure 5:**
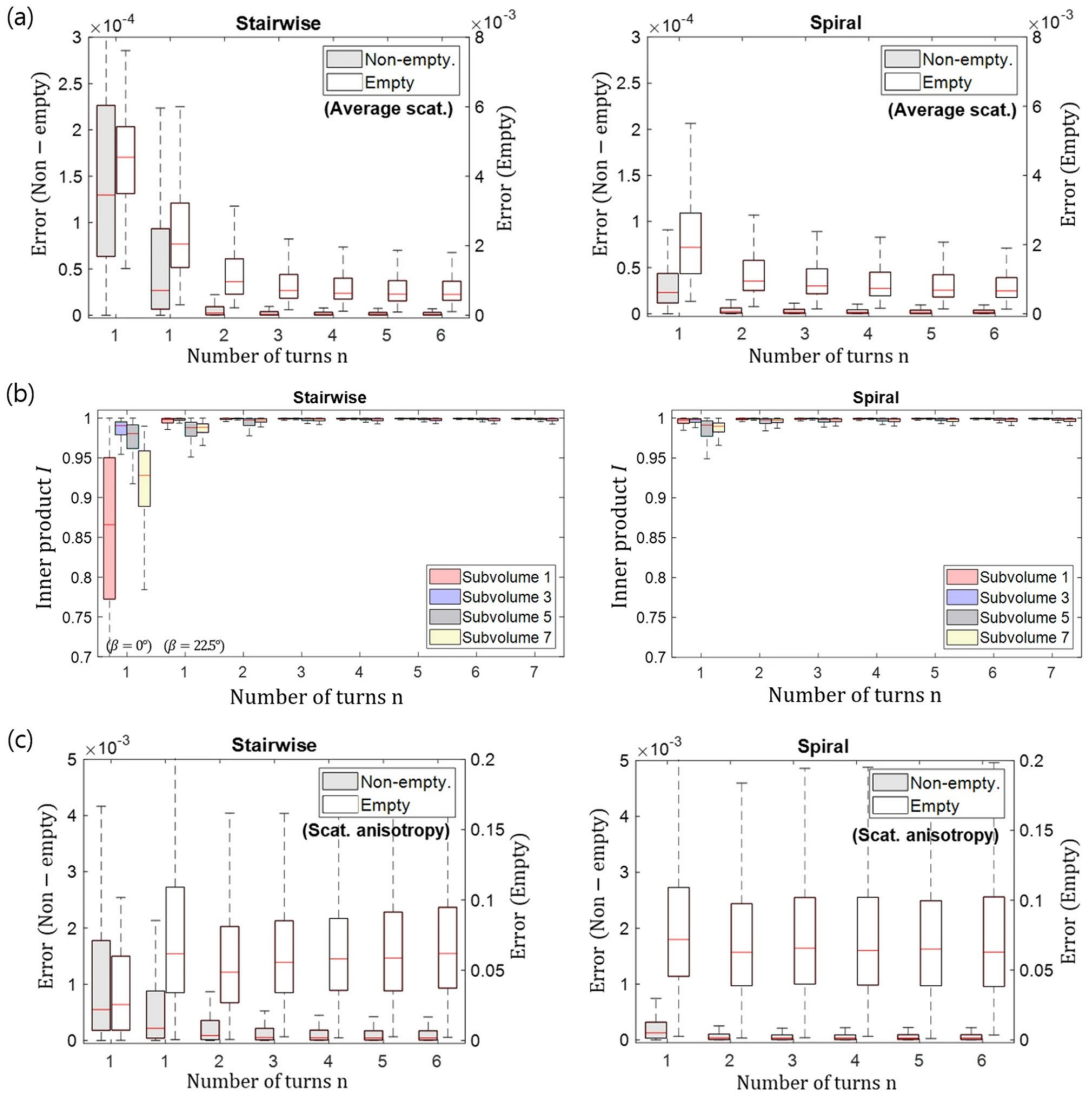
Quantitative assessment of the reconstruction accuracy for the in silico sample. Box plots of the inner product *I* for the different preferential orientations are shown in (**a**). The error *E* of the average scattering is shown in (**b**) and of the scattering anisotropy in (**c**). Note the separate axis scales of the non-empty and the empty volume.

**Figure 7:**
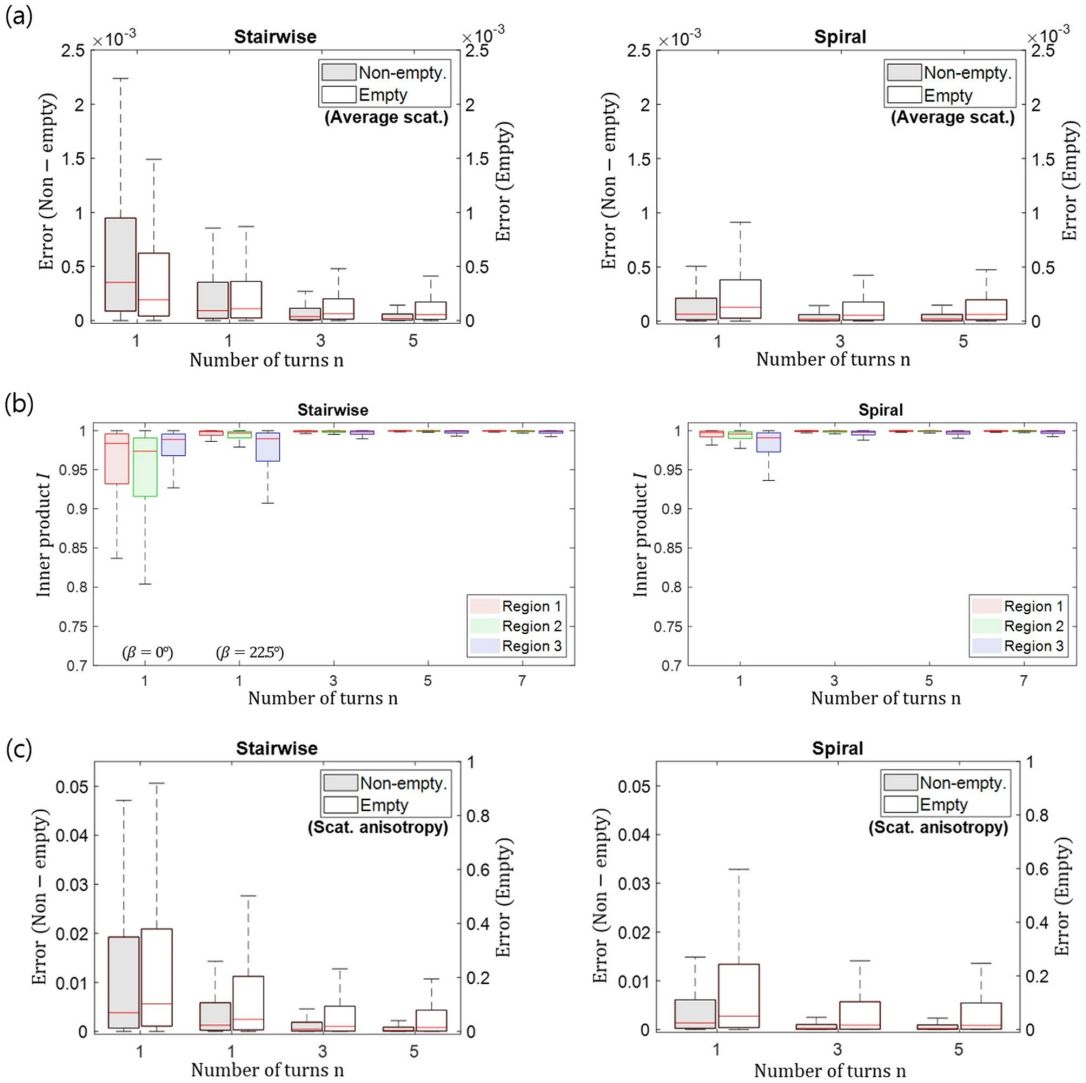
Quantitative assessment of the reconstruction accuracy for the validation sample. Box plots of the inner product *I* for the different preferential orientations are shown in (**a**). The error *E* of the average scattering is shown in (**b**) and of the scattering anisotropy in (**c**). Note the separate axis scales of the non-empty and the empty volume.

In addition, the original version of the Article contained errors in the Introduction and Results section.

In the Introduction,

“X-ray grating interferometry (XGI) based tensor tomography requires an additional angular degrees of freedom (in total three) and thus rather complex acquisition geometry16 because the linear grating do not provide directional scattering sensitivity, leading to a high acquisition overhead.”

now reads:

“X-ray grating interferometry (XGI) based tensor tomography requires additional angular degrees of freedom (in total three) and thus rather complex acquisition geometry16 because the linear grating do not provide directional scattering sensitivity, leading to a high acquisition overhead.”

In the Results section, under the subheading ‘Simulation study’,

“The same analysis has also been performed with only 100 projections (sparse sampling) with the results following the same trend regarding *n* and the accuracy metrics as reported fro 1000 projections.”

now reads:

“The same analysis has also been performed with only 100 projections (sparse sampling) with the results following the same trend regarding *n* and the accuracy metrics as reported from 1000 projections.”

The original Article has been corrected.

