# Supplementary material for: Fast acquisition protocol for X-ray scattering tensor tomography
Source: Sci Rep. 2021 Nov 29;11:23046. doi: 10.1038/s41598-021-02467-w (PMC8629987; doi:10.1038/s41598-021-02467-w)
Supplement: Supplementary file 1 — Supplementary Information. [file 41598_2021_2467_MOESM1_ESM.pdf]

Fast acquisition protocol for X-ray scattering tensor tomography: Supplementary material

Jisoo Kim[1,2], Matias Kagias[2,*,†], Federica Marone[2,*], Zhitian Shi[1,2], and Marco Stampanoni[1,2]

[1]Institute for Biomedical Engineering, University and ETH Zürich, 8092 Zürich, Switzerland
[2]Swiss Light Source, Paul Scherrer Institut, 5232 Villigen, Switzerland
[*]Corresponding authors: mkagias@caltech.edu; federica.marone@psi.ch
[†]Currently at the Division of Engineering and Applied Science, California Institute of Technology, Pasadena, CA 91125, USA

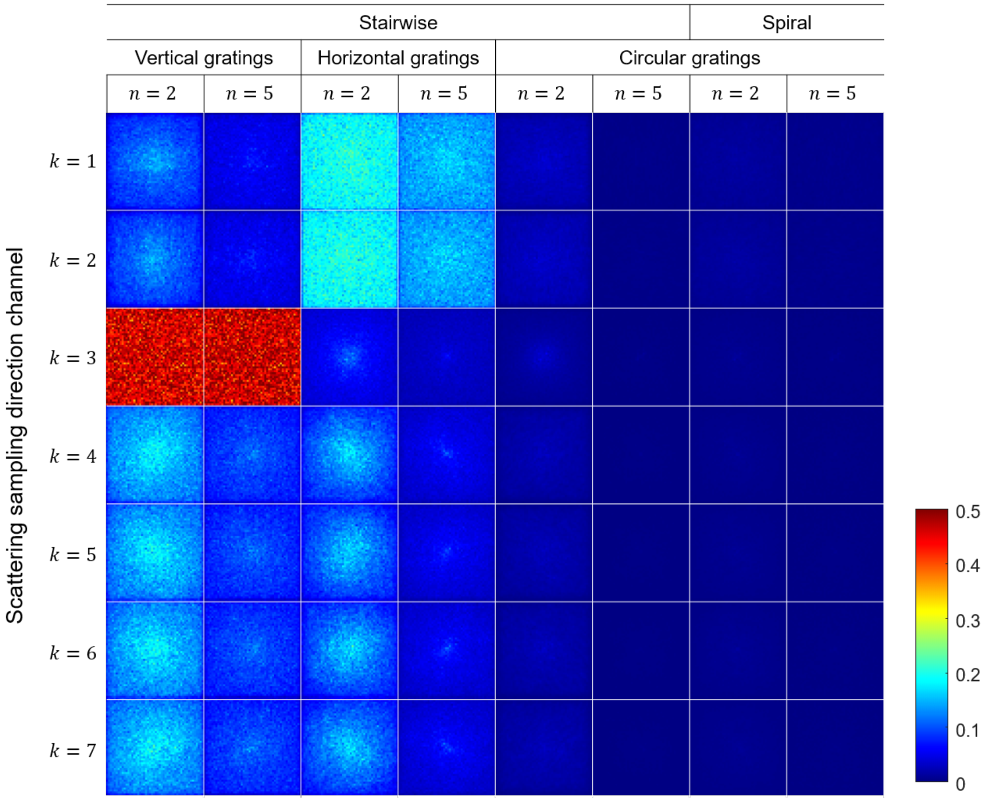

Figure S1. Visualisation of the computed null space for different scattering sampling directions k for the number of turns $n = 2$ and $n = 5$. The null space was computed for different acquisition geometries: stairwise and spiral acquisition; different grating types: vertically aligned linear gratings, horizontally aligned linear gratings, and circular gratings; different number of turns n. High values in null space for scattering component k indicate that the acquisition protocol provides large uncertainties in the reconstruction for that component. The color scale was adjusted to [0, 0.5] to optimize the visual comparison of different acquisition protocols. The figure for the nullspace for $n = 1$ and $n = 10$ is in the main text.

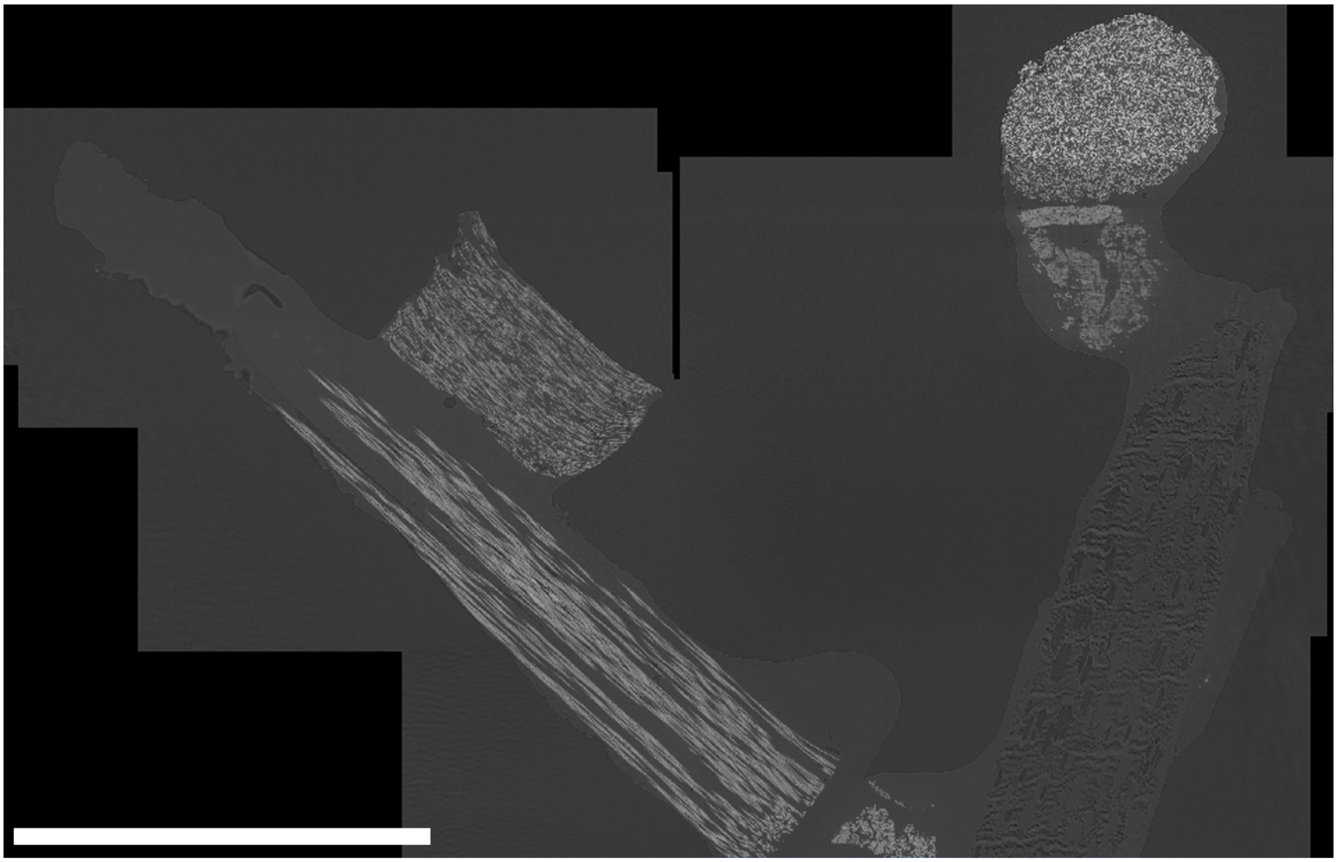

Figure S2. Absorption contrast tomographic slice of the fibre pellet sample reconstructed from projection data measured with a standard X-ray tomographic microscopy approach. This slice approximately corresponds to the slice indicated in Fig. 8c. The pixel size is 11 µm. Numerous local tomography acquisitions were performed to measure the entire sample and the reconstructed volumes were stitched a posteriori[1]. The fibres are aligned along the longer direction of each pellet stick. The scale bar is 5 mm.

References

1. Miettinen, A., Oikonomidis, I. V., Bonnin, A. & Stampanoni, M. NRStitcher: non-rigid stitching of terapixel-scale volumetric images. *Bioinformatics* **35**, 5290–5297, DOI: 10.1093/bioinformatics/btz423 (2019).
